# Health-related quality of life and visual function in retinoblastoma survivors with ocular prostheses: a cross-sectional study

**DOI:** 10.1038/s41598-026-52270-8

**Published:** 2026-05-14

**Authors:** Beatrice Casslén, Richard Jonasson, Marie Odersjö, Ylva Jugård, Kristina Teär Fahnehjelm, Lovisa Lovmar, Marita Andersson Grönlund

**Affiliations:** 1 Department of Clinical Neuroscience, Institute of Neuroscience and Physiology, Sahlgrenska Academy at Gothenburg University, Box 430, 40530 Gothenburg, Sweden; 2 Department of Gynaecology and Obstetrics, Region Västra Götaland, Södra Älvsborg Hospital, Borås, Sweden; 3 Department of Ophthalmology, Region Halland, Halland Hospital Halmstad, Halmstad, Sweden; 4 Department of Otolaryngology, Implant Unit, Region Västra Götaland, Sahlgrenska University Hospital, Gothenburg, Sweden; 5 Department of Ophthalmology, Region Västra Götaland, Södra Älvsborg Hospital, Borås, Sweden; 6 Department of Clinical Neuroscience, Karolinska Institute and Department of Paediatric Ophthalmology, Strabismus and Electrophysiology, St. Erik Eye Hospital, Stockholm, Sweden; 7 Department of Clinical Genetics and Genomics, Region Västra Götaland, Sahlgrenska University Hospital, Gothenburg, Sweden; 8 Department of Ophthalmology, Faculty of Medicine and Health, Örebro University, Örebro, Sweden

**Keywords:** Childhood cancer survivor, Health-related quality of life (HR-QoL), Ocular prosthesis, Perceptual visual dysfunction, Retinoblastoma, Visual function, Visual impairment, Diseases, Health care, Medical research

## Abstract

Retinoblastoma (RB) survivors who have undergone enucleation and use ocular prostheses may face long-term challenges related to visual function and overall well-being. This study aimed to evaluate health-related quality of life (HR-QoL) and visual function in this population, addressing a gap in knowledge regarding their lived experiences and functional vision. This cross-sectional study included 12 unilateral and 3 bilateral RB survivors (10 girls, 5 boys; mean age 15.5 years, range 6.8–26.5), all treated with unilaterally enucleation and an ocular prosthesis at Sahlgrenska University Hospital, Sweden (2000–2019). HR-QoL was assessed using the Paediatric Quality of Life Inventory (PedsQL), both self- and parent-reports. Functional vision was evaluated by best-corrected visual acuity (BCVA) and structured history-taking of perceptual visual dysfunctions (PVDs). Results were compared with age-matched healthy controls. Self-reported PedsQL total score (82.5) equalled normative data (83.0) and parents’ report (82.5). Parents’ reports were comparable to published parental norms (82.5 vs. 87.6). Survivors with subnormal BCVA (≥ 0.2 LogMAR) had significantly lower self-reported HR-QoL scores than those with normal vision (64.1 vs. 88.0, *p* = 0.014), a difference not reflected in parent-reports. Survivors reported more PVDs ≥ 1 area (median 1, range 1–4) than controls (9/15 vs. 1/15, *p* = 0.005). The number of affected PVD areas correlated with age (*r* = 0.59, *p* = 0.022), but not with BCVA or PedsQL score. RB survivors with ocular prostheses require holistic care that includes assessment of HR-QoL and functional vision, beyond visual acuity, to identify unmet needs and guide interventions that support overall well-being and optimal long-term outcomes.

**Supplementary Information:**

The online version contains supplementary material available at 10.1038/s41598-026-52270-8.

## Introduction

Retinoblastoma (RB) is a rare malignant eye tumour of early childhood, with approximately 95% of cases occurring in children under 5 years of age. It accounts for around 6% of all paediatric cancers in this age group, with an annual incidence of 1 in 15,000–18,000 globally^1,2^. The condition originates from retinal mutations in the recessive *RB1* tumour-suppressor gene. About 60% of patients develop non-heritable, unilateral RB from somatic biallelic mutations in a single retinal cell, while the remaining 40% have inherited, typically bilateral RB, with a constitutional *RB1* mutation in all cells and a second somatic mutation in a retinal cell. Inherited cases predispose patients to multiple intraocular tumours and other malignancies, such as sarcomas, melanomas, leukaemia, and brain tumours^2–4^. Treatment for RB has evolved greatly during the 20th century, from systemic chemotherapy to targeted modalities such as external beam radiotherapy, ophthalmic artery chemosurgery, and intravitreal injections, substantially reducing the need for enucleation from 95% to under 10%. Nevertheless, enucleation remains necessary for advanced tumours or when all visual function is lost^5^.

Beyond vision loss, the removal of an eye in early childhood may result in facial deformities due to disrupted orbital development, potentially affecting psychosocial well-being and health-related quality of life (HR-QoL). To mitigate these effects, patients typically receive an orbital implant and prosthetic eye, which when fitted in childhood, supports orbital growth, preserves facial symmetry, and contributes to improved HR-QoL^6^. Although HR-QoL is an important outcome in RB survivorship, existing data are limited and inconsistent. Some studies suggest reduced HR-QoL, while others report levels comparable to healthy controls or to normative data, highlighting the complex interplay of medical, functional, and psychosocial factors shaping survivor well-being^7–10^. To optimize long-term support and outcomes for children and young adults with RB, it is essential to explore beyond standard clinical indicators. Impaired visual acuity (VA) has been shown to negatively affect HR-QoL among RB survivors, but broader aspects of functional vision remain less frequently studied^7,11,12^. Because VA represents only one dimension of functional vision, complementary assessments are needed to capture how vision impairments affect daily life. Evaluating functional vision beyond VA may provide a more complete understanding of the challenges faced by this population.

One such aspect is the presence of perceptual visual dysfunctions (PVDs), also referred to as cognitive visual disorders, which arise from atypical processing within the brain’s visual networks and influence how visual information is interpreted and used in everyday activities. These disturbances may result from disrupted interactions among cortical regions, leading to difficulties with visuospatial processing, visually guided behaviour, and object or face recognition. PVDs are commonly grouped into five functional domains: orientation (O), recognition (R), depth perception (D), movement perception (M), and simultaneous perception/crowding (S), each reflecting distinct aspects of functional vision critical for everyday activities and daily functioning^13–15^. Such dysfunctions can interfere with visual-based daily activities even when VA and visual fields are normal or near normal. PVDs are a core feature of cerebral visual impairment (CVI), a broader clinical condition in which visual dysfunction result from damage or atypical development of the brain rather than the eyes. While CVI encompasses a spectrum of neurological visual impairments, PVDs specifically describe the functional consequences on perception and the use of visual information in everyday life. PVDs are commonly found in children with prematurity, hydrocephalus or neurodevelopmental disorders, and often overlap with psychological conditions like autism, learning disability and attention deficit hyperactivity disorder^13,14,16,17^. Despite their clinical relevance in other groups, the impact of PVDs in RB survivors has not, to our knowledge, previously been investigated. To address this knowledge gap, we investigated PVDs and HR-QoL in relation to visual function among RB survivors (both unilateral and bilateral) wearing unilateral ocular prostheses. Our aim was to provide a more comprehensive understanding of their visual and psychosocial challenges, thereby contributing to the development of tailored, long-term care strategies.

## Methods

### Study design and participants

This study aimed to assess HR-QoL and PVDs in RB survivors treated with ocular prostheses. A combined retrospective and prospective cross-sectional cohort design was employed. The retrospective component involved a review of medical records to gather clinical data. The prospective component included validated HR-QoL questionnaires completed by RB survivors and their parents, as well as structured interviews to assess the presence of PVDs. Results were compared with normative data and healthy controls^18^.

Between 2000 and 2019, a total of 27 individuals treated for RB were each fitted with a handmade ocular prosthesis by the same anaplastologist (M.O.) at the Department of Otolaryngology, Implant Unit, Sahlgrenska University Hospital in Gothenburg, Sweden. Due to the rarity of RB, no formal a priori sample size calculation was feasible; instead, the study was designed to include all eligible individuals during the study period.

All children and young adults with RB at this hospital were invited to participate in this study; two declined, one was no longer registered in the Swedish population register, seven did not respond, and two initially accepted but did not respond thereafter. Hence, 15 individuals treated for RB (unilateral or bilateral) and wearing unilateral ocular prosthesis were enrolled in the current study. Participants were excluded if they were unable to understand Swedish, as this was required for questionnaire completion and the structured history taking.

The participants had an age range from 6.8 to 26.5 years, representing a broad age span. All assessments were, however, conducted and interpreted using age-appropriate methods. The PedsQL questionnaire was applied according to validated age-specific norms, PVD controls were matched for age and gender, and best-corrected visual acuity (BCVA) was measured with age-appropriate methods. This approach aimed to account for developmental differences across the age range.

### Medical record review

Medical records of all patients were collected from Sahlgrenska University Hospital in Gothenburg, St. Erik Eye Hospital in Stockholm, and the local hospitals in Region Västra Götaland where any of the patients may have been treated and/or followed up. The data covered: tumour laterality (unilateral or bilateral), affected eye side (right eye [RE], left eye [LE], or RE + LE), treatment with chemotherapy and radiation, genetic screening, number of ophthalmological surgeries, age when treated with an ocular prosthesis, number of prosthesis-associated infections and other diagnoses including but not limited to secondary malignancies.

BCVA was retrieved from medical records at the measurement occasion closest to questionnaire completion by patients and parents. The assessment method was selected according to each participant’s age and functional capacity, specifically using KM charts^19^. BCVA was initially recorded in decimal notation and subsequently converted to logarithm of the minimum angle of resolution (LogMAR) for statistical analyses, where 0.0 represents normal VA and higher values indicate poorer vision.

### Health-related quality of life assessment

The Paediatric Quality of Life Inventory (PedsQL) 4.0 is a 23-item questionnaire measuring HR-QoL across four domains: physical, school, social, and emotional functioning. The latter three domains are combined into a psychosocial health score, while a total score reflects all 23 items. Self-report and parent-report versions are adapted for the following age groups: 2–4, 5–7, 8–12, 13–18 (teens), 18–25 (young adults), and > 26 years (adults). Self-reports are available from ≥ 5 years. Responses are rated on a five-point Likert scale, ranging from 0 (never a problem) to 4 (almost always a problem). Scores are reverse-scored and transformed: 0 = 100, 1 = 75, 2 = 50, 3 = 25, and 4 = 0. Final scores are averaged per domain and for the total scale. Higher scores indicate better HR-QoL. Age-specific questionnaires were distributed to all RB survivors and their parents^18–20^.

### Perceptual visual dysfunction assessment

The information about perceptual visual dysfunctions (PVDs) was collected through structured history-taking with caregivers of children aged ≥ 3 years and from the children/young adults themselves when possible. The structured history-taking used in this study is a shortened version of the questionnaire developed by Dutton et al.^21,22^ and is applied at our clinic as part of the clinical evaluation for CVI. The history-taking consists of 12 questions, each targeting one of five domains of visual perceptual difficulties: recognition (questions 1–4), orientation (questions 5–7), depth perception (question 8), movement perception (questions 9 and 10), and simultaneous perception (questions 11 and 12; difficulty identifying objects within visually complex or cluttered scenes). Each question required a yes/no response and was designed to identify the presence of PVDs, the number of affected domains, and the specific area(s) impacted^22,23^. The questionnaire used to assess PVDs is available in Supplementary material.

### Normative data and control group

The PedsQL self- and parent-report results were compared with previously published normative data from both child and parent assessments^18^. For comparisons of PVD, all cases were matched to controls by sex and age (± 2 years of decimal age)^24^. Controls were recruited from typically developing children and young adults attending local schools in the same Swedish region. From an initial pool of 204 healthy children and young adults, 15 individuals (10 girls, 5 boys; mean age 15.3 years, range 5–27 years) were selected to ensure comparability with the RB cohort.

### Statistical analysis

Descriptive statistics, including means, standard deviations (SD), medians, and ranges, were calculated to summarize the data. PedsQL-4.0 scores were analysed using the scaling and scoring manual by Varni et al.^18–20^. To compare the means between the RB group and normative data, a two-sided Student’s *t*-test was used. For comparison between self-reports and parent reports, Fisher’s non-parametric permutation test for matched pairs was used. For comparison of HR-QoL within the groups according to BCVA, Fisher’s non-parametric permutation test was used for continuous variables. To compare the presence of PVD between the RB group and healthy controls, Fisher’s exact test was used. Spearman correlation coefficients were employed to assess the relationships between the PedsQL total score and BCVA, as well as the number of PVD areas. Additionally, Spearman correlation coefficients were also used to examine associations between the number of PVD areas, BCVA, and age. Statistical significance was indicated by *p* values < 0.05.

## Results

### Participants characteristics

The study included 15 individuals (10 girls, 5 boys) with a mean age of 15.5 years (range 6.8–26.5). Three had bilateral RB and 12 had unilateral disease; all were treated unilaterally with enucleation and fitted with an ocular prosthesis. Mean age at first fitting with an ocular prosthesis was 2.9 years (range 0.8–7.5 years). Additional details about the study population, including treatment history and associated diagnoses, are presented in Table 1.

**Table 1:** Clinical characteristics of the study population with retinoblastoma treated with enucleation and an ocular prosthesis.

Ref. no./Age (years)/ Sex	Unilateral/Bilateral disease	Follow-up time (years)	Age when treated with an ocular prosthesis (years)	Side of ocular prosthesis	Treated with chemo-therapy	Treated with radiation therapy	No. of ophthalmo-logical surgeries	No. of prosthesis-associated infections	Other associated diagnoses	Genetic analysis
#1/7.8/F	Bilateral	6.1	1.7	Left	Yes (both eyes)	Yes (right eye)*	2	4	No	NA
#2/10.1/M	Unilateral	2.6	7.5	Left	No	No	1	1	No	NA
#3/11.9/F	Unilateral	10.1	1.8	Left	No	No	3	1	Asthma	NA
#4/17.3/F	Unilateral	15.5	1.8	Left	No	No	2	2	No	NA
#5/18.8/M	Unilateral	18.0	0.8	Right	No	Yes (right eye)	4	2	Partial monosomy 13q, delayed psychomotor development, growth hormone treated	NA
#6/21.7/F	Bilateral	18.5	3.2	Left	Yes (right eye)	Yes (right eye)	6	3	Cataract, asthma, anxiety	NA
#7/21.9/F	Unilateral	17.6	4.3	Right	No	No	5	3	No	NA
#8/22.1/F	Bilateral	20.9	1.2	Right	No	Yes (left eye)	4	3	Osteosarcoma femur, treated with surgery, chemotherapy, and limb prosthetics; lung metastasis, treated with partial lung resection and chemotherapy	NA
#9/25.6/M	Unilateral	24.1	1.5	Left	No	No	1	0	No	NA
#10/26.0/F	Unilateral	24.0	2.0	Left	No	No	3	6	No	No mutation identified
#11/12.0/F	Unilateral	8.0	4.0	Right	No	No	1	9	Anxiety	NA
#12/10.7/M	Unilateral	10.0	2.7	Right	No	No	3	7	No	No mutation identified
#13/7.8/F	Unilateral	5.5	2.2	Right	No	No	1	0	No	Homozygote deletion in RB1
#14/12.0/M	Unilateral	6.1	5.9	Left	Yes (left eye)**	NA	1	0	No	Heterozygote deletion of exon 9 in RB1
#15/6.8/F	Unilateral	3.7	3.1	Right	Yes (right eye)	No	1	1	No	No mutation identified

Clinical characteristics of the study population, comprising 15 children and young adults with retinoblastoma, all treated with enucleation and fitted with an ocular prosthesis, including follow-up duration, age at prosthesis fitting, treatment history, ocular complications, associated diagnoses, and results from available genetic analyses. F = female; M = male; NA = not answered; RB = Retinoblastoma; Ref. no. = reference number. *Also treated with cryo- and brachytherapy. **Treatment abroad, documentation unavailable.

### Health-related quality of life findings

Thirteen of 15 RB survivors in our study group (9 girls, 4 boys; mean age 16.4 years, range 6.8–26.5 years) completed the PedsQL questionnaire, reporting HR-QoL scores comparable to normative data (Table 2). Parents of RB survivors (*n* = 15) reported their child’s QoL as comparable to that of parents of healthy children/young adults (Table 2). No statistically significant differences were found between survivor self-reports and parent-reports (*n* = 12; note that one of the 13 individuals with RB lacked a completed parental report) (Table 3).

**Table 2:** Health-related quality of life in retinoblastoma survivors and their corresponding parent, compared with normative data.

HR-QoL	Child/young adult with RB (*n* = 15)*	Child/young adult (Normative data) (*n* = 401)*	*P* value
PedsQL, total			
Mean (SD)	82.52 (12.46) (*n* = 13)	83.00 (14.79)	0.91
PedsQL, physical health			
Mean (SD)	89.18 (13.40) (*n* = 13)	84.41 (17.26) (*n* = 400)	0.33
PedsQL, psychosocial			
Mean (SD)	78.97 (13.03) (*n* = 13)	82.38 (15.51) (*n* = 399)	0.43
Emotional	77.69 (18.46) (*n* = 13)	80.86 (19.64) (*n* = 400)	0.57
Social function	87.69 (15.27) (*n* = 13)	87.42 (17.18) (*n* = 399)	0.96
School function	71.54 (15.11) (*n* = 13)	78.63 (20.53) (*n* = 386)	0.22

	Parent (RB) (*n* = 15)*	Parent (Normative data) (*n* = 717)*	*P* value
PedsQL, total			
Mean (SD)	82.52 (18.41) (*n* = 13)	87.61 (12.33)	0.15
PedsQL, physical health			
Mean (SD)	87.74 (21.42) (*n* = 13)	89.32 (16.35)	0.73
PedsQL, psychosocial			
Mean (SD)	79.75 (17.24) (*n* = 13)	86.58 (12.79)	0.058
Emotional	75.77 (18.28) (*n* = 13)	82.64 (17.54)	0.16
Social function	86.54 (19.84) (*n* = 13)	91.56 (14.20) (*n* = 716)	0.21
School function	80.00 (18.60) (*n* = 12)	85.47 (17.61) (*n* = 611)	0.29

This table presents HR-QoL scores from the PedsQL questionnaire for 13 of 15 age-eligible children and young adults with retinoblastoma, and their corresponding parents. Results are compared with normative published data. Self-reports were available for participants aged ≥ 5 years; parent-reports were available for parents of children aged 1–18 years. Mean (SD) scores are shown, with higher values indicating better HR-QoL. Differences between groups were calculated using a two-sided Student’s t-test. HR-QoL = health-related quality of life; PedsQL = Paediatric Quality of Life Inventory; RB = Retinoblastoma; SD = standard deviation. *Where values differ between child and parent reports within the same group, data are presented separately.

**Table 3:** Paired comparison of self- and parent-reported health-related quality of life using PedsQL in retinoblastoma survivors.

HR-QoL	Child/young adultwith RB (*n* = 12)	Parent (*n* = 12)*	Mean difference between groups (95% CI)	*P* value
PedsQL, total				
Mean (SD)	82.34 (12.95)	81.88 (19.03)	0.46 (12.68)	0.94
Median (range)	86.96 (55.43; 95.65)	90.22 (41.24; 100.0)	–1.09 (–14.14; 33.7) (–6.52; 8.71)	
PedsQL, physical health				
Mean (SD)	88.80 (13.88)	86.98 (22.13)	1.82 (14.81)	0.72
Median (range)	96.88 (62.50; 100.0)	100 (40.63; 100.0)	0 (–25; 34.38) (–6.87; 11.25)	
PedsQL, psychosocial				
Mean (SD)	78.89 (13.56)	79.17 (17.83)	–0.28 (12.41)	0.92
Median (range)	81.67 (51.67; 93.33)	85.0 (41.67; 100.0)	–1.67 (–18.34; 33.33) (–7.00; 7.67)	
Emotional	77.92 (19.20) 85.0 (25.0; 100.0)	75.42 (18.98) 80.0 (30.0; 100.0)	2.50 (12.15) 0 (–10; 35) (–4.00; 10.00)	0.66
Social function	87.08 (15.74) 90.0 (40.0; 100.0)	85.83 (20.50) 97.5 (40.0; 100.0)	1.25 (14.94) 0 (–20; 30) (–7.50; 11.00)	0.87
School function	71.67 (15.72) 72.50 (45.0; 100.0)	79.55 (19.36) 85.0 (45.0; 100.0) (*n* = 11)	–5.45 (16.50) –10 (–30; 35) (–15.00; 6.25)	0.34

PedsQL self-reports were available for children aged ≥ 5 years, and parent-reports were available for parents of children aged 1–18 years. Higher scores indicate better quality of life. Fisher’s non-parametric permutation test for matched pairs was used to compare scores within groups. The 95% confidence interval (CI) for the mean was calculated using inversion of the same test. CI = confidence interval; PedsQL = Paediatric Quality of Life Inventory; SD = standard deviation. *Where values differ between child and parent reports within the same group, data are presented separately.

No association was found between PedsQL total score and BCVA. However, when RB survivors were categorized by their VA status, i.e., subnormal BCVA (≥ 0.2 LogMAR) vs. normal BCVA, those with subnormal BCVA (*n* = 4) showed lower HR-QoL. Interestingly, this impact was not perceived by parents (Table 4). Out of the four participants categorized as having subnormal VA, three were treated for bilateral RB and met the World Health Organization (WHO) criteria for visual impairment (VI); VA ≥ 0.5 LogMAR in the better eye (Table 5 ref. #1, #6, and #8).

**Table 4:** Health-related quality of life in RB survivors with normal vs. subnormal vision and their parents.

HR-QoL	RB survivors Normal VA VA < 0.2 LogMAR (*n* = 11)*	RB survivors Subnormal VA VA ≥ 0.2 LogMAR (*n* = 4)*	Mean difference between groups (95% CI)	P-value
PedsQL, total				
Mean (SD)	88.0 (5.8)	64.1 (14.1)	23.9	**0.014**
Median (range)	87 (76.1; 95.7) (*n* = 10)	56.5 (55.4; 80.4) (*n* = 3)	(8.7; 38.0)	
PedsQL, physical health				
Mean (SD)	93.8 (9.7)	74.0 (17.2)	19.8	**0.049**
Median (range)	100 (75; 100) (*n* = 10)	65.6 (62.5; 93.8) (*n* = 3)	(3.1; 35.9)	
PedsQL, psychosocial				
Mean (SD)	85.0 (6.0)	58.9 (12.5)	26.1	**0.007**
Median (range)	84.2 (76.7; 93.3) (*n* = 10)	51.7 (51.7; 73.3) (*n* = 3)	(15.0; 40.0)	
Emotional	83.5 (11.8) 87.5 (60; 100) (*n* = 10)	58.3 (29.3) 70 (25; 80) (*n* = 3)	25.2 (–2.5; 60.0)	0.10
Social function	93.5 (6.3) 95 (80; 100) (*n* = 10)	68.3 (24.7) 80 (40; 85) (*n* = 3)	25.2 (5.0; 50.0)	**0.021**
School function	78.0 (10.6) 77.5 (65; 100) (*n* = 10)	50.0 (8.7) 45 (45; 60) (*n* = 3)	28.0 (15.0; 45.0)	**0.007**

	Parents of survivors with normal VA VA < 0.2 LogMAR (*n* = 11)*	Parents of survivors with subnormal VA VA ≥ 0.2 LogMAR (*n* = 4)*	Mean difference between groups, (95% CI)	*P* value
PedsQL, total				
Mean (SD)	87.8 (14.0)	70.6 (25.9)	17.2	0.20
Median (range)	90.2 (52.2; 100) (*n* = 9)	73.4 (41.3; 94.6)	(–4.3; 44.6)	
PedsQL, physical health				
Mean (SD)	93.1 (18.6)	75.8 (28.1)	17.3	0.23
Median (range)	100.0 (43.8; 100) (*n* = 9)	81.3 (40.6; 100)	(–10.9; 46.9)	
PedsQL, psychosocial				
Mean (SD)	85.0 (12.2)	67.9 (25.0)	17.1	0.16
Median (range)	85.0 (56.7; 100) (*n* = 9)	69.2 (41.7; 91.7)	(–5.8; 43.3)	
Emotional	80.0 (15.6) 85.0 (55; 100) (*n* = 9)	66.3 (25.0) 75.0 (30; 85)	13.8 (–13.3; 40.0)	0.30
Social	92.8 (13.0) 100 (60; 100) (*n* = 9)	72.5 (29.6) 75.0 (40; 100)	20.3 (–5.0; 50.0)	0.16
School function	82.2 (18.6) 85.0 (45; 100) (*n* = 9)	73.3 (24.7) 85.0 (45; 90) (*n* = 3)	8.89 (–25.00; 40.0)	0.57

The PedsQL was completed by individuals with RB treated with ocular prostheses and divided based on BCVA) in the fellow eye: normal or subnormal (≥ 0.2 LogMAR). Parent-reports were similarly grouped according to their child’s visual acuity. PedsQL self-reports were available for children aged ≥ 5 years and parent-reports were available for parents of children aged 1–18 years. Higher mean scores indicate better quality of life. Bold indicates statistical significance. For comparison between groups, Fisher’s non-parametric permutation test was used for continuous variables. The 95% confidence interval (CI) for the mean was calculated using inversion of the same test. BCVA = best-corrected visual acuity; CI = confidence interval; HR-QoL = health-related quality of life LogMAR = logarithm of the minimum angle of resolution; PedsQL = Paediatric Quality of Life Inventory; RB = Retinoblastoma; SD = standard deviation. * Where the numbers differ between children and parents in the group, they are reported separately for each category. Significant values are in [bold].

**Table 5:** Summary of 15 children/young adults with retinoblastoma (RB), treated with enucleation and unilateral ocular prosthesis: demographics, uni- or bilateral RB, best-corrected visual acuity (BCVA), presence of perceptual visual dysfunctions (PVDs), total and school health-related quality of life (HR-QoL) using PedsQL questionnaire, including both self- and parent-reports.

Ref. no./ Age (years)/ Sex	RB Unilateral/Bilateral	BCVA LogMAR	PVD Yes/No	PVD Number of PVDs	PVD Affected area(s)	PedsQL Total score, self-report	PedsQL School function, self-report	PedsQL Total score, parent report	PedsQL School function, parent report
#1/7.8/F	Bilateral	0.6	No	–	–	NA	NA	90.22	85.00
#2/10.1/M	Unilateral	0.0	Yes	1	S	85.87	80.00	52.17	45.00
#3/11.9/F	Unilateral	0.1	Yes	2	D, Mo	95.65	90.00	100.00	100.00
#4/17.3/F	Unilateral	0.0	No	–	–	89.13	65.00	90.22	65.00
#5/18.8/M	Unilateral	0.4	Yes	2	D, Mo	56.52	45.00	56.52	45.00
#6/21.7/F	Bilateral	0.7	Yes	2	D, S	80.43	60.00	94.57	90.00
#7/21.9/F	Unilateral	0.0	Yes	1	D	86.96	80.00	90.22	100.00
#8/22.1/F	Bilateral	1.0	Yes	4	R, O, D, S	55.43	45.00	46.25	55.00
#9/25.6/M	Unilateral	-0.3	Yes	1	S	86.96	80.00	94.57	90.00
#10/26.0/F	Unilateral	-0.10	Yes	1	D	84.78	70.00	NA	NA
#11/12.0/F	Unilateral	-0.10	No	–	–	76.09	70.00	88.04	85.00
#12/10.7/M	Unilateral	-0.10	No	–	–	92.39	75.00	93.48	75.00
#13/7.8/F	Unilateral	0.0	No	–	–	95.65	100.00	95.65	100.00
#14/12.0/M	Unilateral	0.0	Yes	1	S	NA	NA	NA	NA
#15/6.8/F	Unilateral	0.10	No	–	–	86.96	70.00	85.87	80.00

Demographics, best-corrected visual acuity (BCVA), presence of perceptual visual dysfunctions (PVDs) and areas affected, and Paediatric Quality of Life Inventory (PedsQL) scores (total and school-related), including both self- and parent-reports among all 15 RB survivors in the study group. PedsQL self-reports were available for children aged ≥ 5 years and parent-reports were available for parents of children aged 1–18 years. PedsQL total score represents the mean of all 23 items answered. BCVA = best-corrected visual acuity; D = depth perception; F = female; LogMAR = logarithm of the minimum angle of resolution; M = male; Mo = movement perception; NA = not answered; O = orientation; PedsQL = Paediatric Quality of Life Inventory; PVDs = perceptual visual dysfunctions; R = recognition; RB = Retinoblastoma; Ref. no. = reference number; S = simultaneous perception; – = not applicable.

### Perceptual visual dysfunctions findings

**Fig. 1:**
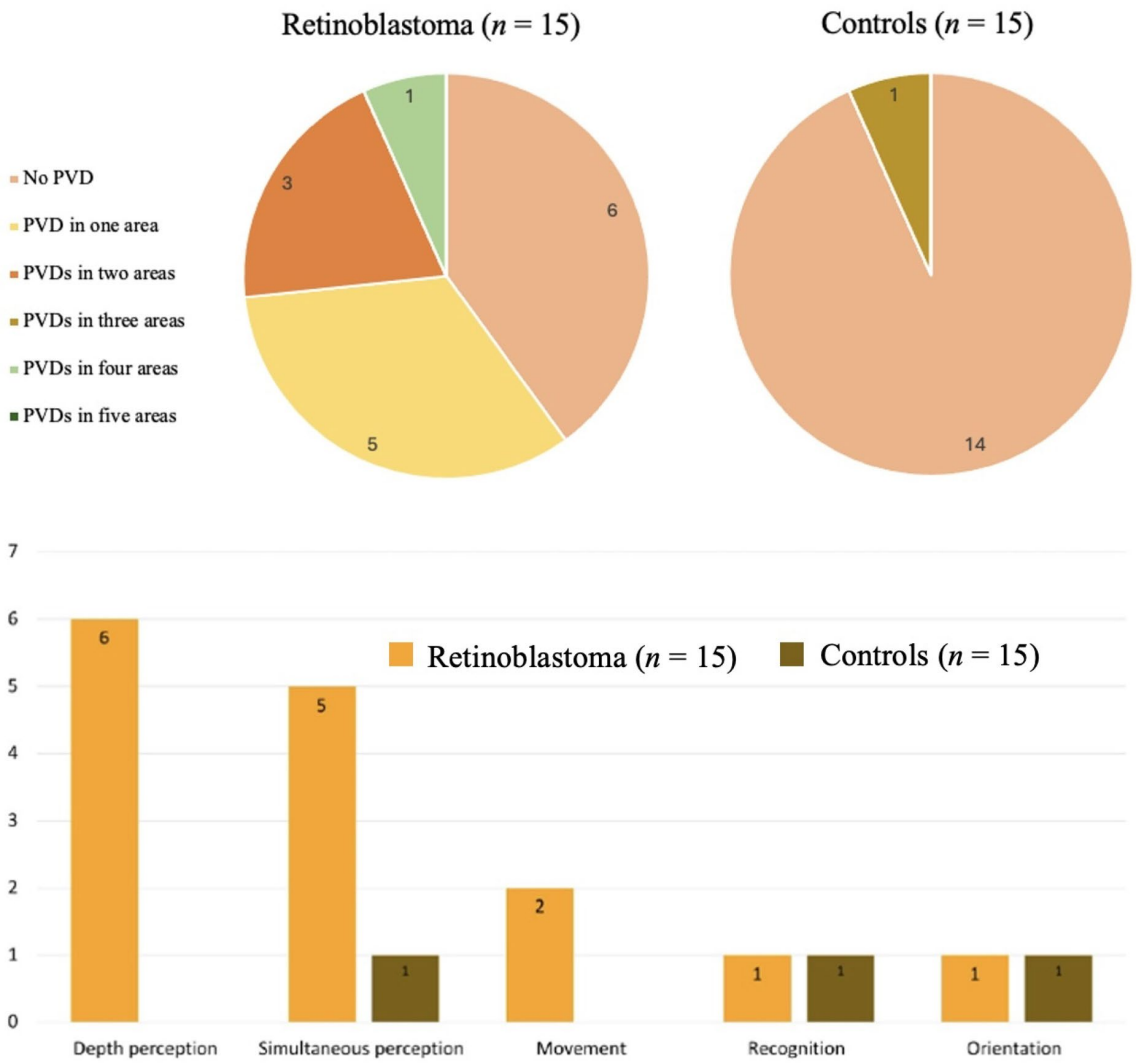
Perceptual visual dysfunctions (PVDs) reported by retinoblastoma survivors (*n* = 15) and healthy controls (*n* = 15). The top panel shows the number of perceptual visual dysfunction (PVD) areas affected, as reported by retinoblastoma (RB) survivors treated with an ocular prosthesis (*n* = 15) and by healthy controls (*n* = 15). The bottom panel displays the number of individuals reporting dysfunction in each of the five PVD areas, comparing RB survivors and controls. Each bar represents one PVD area, and the height indicates the number of individuals reporting dysfunction in that specific area (ranging from 0 to 6). The five PVD areas assessed were: depth perception, simultaneous perception, movement perception, recognition, and orientation perception. Participants could report difficulties in more than one area. These data illustrate the distribution and frequency of PVDs among RB survivors relative to healthy controls.

More RB survivors (9/15) reported PVDs in one or more areas (median 1, range 1–4) than did healthy controls (1/15) (*p* = 0.005, Fisher’s exact test), (Fig. 1, top). Excluding individuals with RB who reported PVD in only depth perception (*n* = 2), RB survivors still reported more PVDs than did healthy controls (*p* = 0.011). Similar, when excluding those with PVDs in only simultaneous perception (*n* = 3; difficulty identifying objects within visually complex or cluttered scenes), RB survivors continued to report significantly more PVDs than controls (*p* = 0.024). For all other PVD areas, difficulties were reported in combination with problems in other domains (Table 5). We found a positive association between number of affected PVD areas and age (*r* = 0.59, *p* = 0.022, Spearman correlation coefficients), indicating that older participants reported more difficulties. However, no association between number of PVD areas and BCVA or PedsQL total score was found. Individuals with RB and healthy controls who reported PVDs in each of the five areas (i.e., depth, simultaneous, movement, recognition, and orientation perception) are shown in Fig. 1 (bottom).

Table 5 presents information on all 15 individuals with RB including age, gender, uni- or bilateral involvement, BCVA, presence of PVDs, total and school-related scores from the PedsQL inventory, as assessed by both self- and parent-reports.

## Discussion

This study addressed an important gap in current research through evaluation of HR-QoL in relation to functional vision, including PVDs, among enucleated children and young adults with RB who received ocular prosthesis treatment. Existing research on HR-QoL among RB survivors and their parents has generated varied results, potentially influenced by factors such as assessment tools, treatments, vision, age at diagnosis, and demographic heterogeneity^7,25,26^. By assessing HR-QoL and PVDs among enucleated RB survivors from both the survivors’ and parents’ perspectives, we provided valuable insights into their well-being and functional vision challenges.

In the present study, RB survivors and their parents reported HR-QoL comparable to population norms, consistent with findings from previous research^8,10,27^. This is consistent with a review by Belson et al., demonstrating that only 5 of 15 studies reported a decrease in HR-QoL when compared to controls or normative data, while also emphasizing the need for further research to clarify factors influencing HR-QoL^25^. Although not statistically significant, our results revealed a pattern whereby RB survivors reported lower HR-QoL scores in the psychosocial, school and emotional dimensions than did healthy controls. This is consistent with previous studies identifying physical, social, emotional, and school functioning, as specific areas affected, despite an overall good HR-QoL^10,27,28^. However, Batra and co-workers found RB survivors to experience preserved physical functioning^29^ and Dijk et al. reported better HR-QoL on the dimensions “moods and emotions” and “autonomy compared to controls”^8^.

While the self- and parent-reported HR-QoL scores in our study were largely comparable, parents tended to report slightly lower scores in the emotional, social, and physical domains; however, these differences were not statistically significant. This finding is consistent with previous research, in which parents rated their child’s HR-QoL lower than the survivors themselves, particularly in these domains^7,8,10,26^. In contrast, Simeonov et al. and Arteaga Henríquez et al. found that RB survivors reported slightly lower QoL scores than their parents, although these differences were also not statistically significant^30,31^. These discrepancies highlight the importance of including both RB survivor and parent perspectives when assessing HR-QoL, as each may provide unique and complementary insights into the RB survivor’s well-being.

Despite our limited number of participants, subnormal BCVA compared with normal BCVA, generated significantly reduced HR-QoL scores across all domains except emotional functioning. The most pronounced effects were observed in psychosocial and school-related functioning, highlighting the broader impact of visual function on daily life and school settings. Interestingly, parents did not perceive this impact on their children’s HR-QoL, suggesting a potential gap in awareness of the child’s functional vision difficulties that could delay necessary support. This divergence could reflect factors such as the children’s immediate struggles versus the parents’ broader perspective of well-being. Therefore, it is essential that clinicians actively discuss the day-to-day visual challenges with survivors and their families during follow-up visits to guide appropriate interventions.

Consistent with our findings, previous studies on RB have linked VI to absenteeism and reduced school-related QoL, with contributing factors including social stigma, frequent hospital visits, and learning difficulties^7,9,12,27,29,31^. Similar patterns have been reported in children and young adults with other ophthalmic or neurodevelopmental conditions, that may result in poor vision, such as nystagmus, anophthalmia/microphthalmia (A/M), albinism, hydrocephalus and Fetal Alcohol Spectrum Disorder (FASD), who also experience reduced HR-QoL compared with peers without visual disability^16,32^. Moreover, Dhingra et al. found that survivors often struggled to keep up in school and reported memory difficulties, suggesting that both visual and cognitive factors may contribute to academic challenges^7^. Collectively, these findings identify school-related functioning as one of the domains most affected in survivors with subnormal BCVA. This underscores the importance of raising awareness among parents and teachers about the impact of visual function, to enable tailored support and accommodations that promote both general and school-related well-being for RB survivors and other groups with subnormal BCVA.

Enucleated survivors reported to experience significant difficulties in daily functioning, including reduced motor function, limiting participation in daily-, and school activities, despite normal vision in the fellow eye^9^. Similar, Barnett et al. found that RB survivors exhibited reduced reading acuity and slower reading speeds despite preserved vision, supporting the view that VA alone does not fully capture functional vision^33^. In a cross-sectional study, Reynolds et al. reported that ten out of eleven RB survivors exhibited impaired saccadic eye movements and decreased contrast sensitivity, underscoring that standard measures like VA may underestimate the true extent of visual dysfunctions faced by RB survivors^34^. These visual functioning deficits, including visual field loss, impaired saccades, and poor contrast sensitivity, have been associated with diminished daily functioning and lower HR-QoL among RB survivors, even in the presence of normal acuity^11,34^. Although some studies have focused on these specific aspects of visual function beyond VA, yet little is known about how RB survivors perceive, interpret and process visual information, i.e., PVDs. This gap is critical, as PVDs may more accurately reflect a comprehensive understanding of the impact of functional vision on daily life and well-being. A key contribution of this study is therefore the focus on PVDs.

In the current study, the prevalence of PVDs was significantly higher among survivors with ocular prostheses than controls, irrespective of BCVA. This is consistent with previous research showing that the presence of PVDs is not necessarily associated with VA^13,35,36^. Further, individuals with diagnosis such as anophthalmia/microphthalmia, hydrocephalus and FASD have also been reported to exhibit more PVDs compared with healthy controls^16,37^. When comparing PVDs in our current cohort of RB survivors with those observed in our previous study of A/M^36^, no significant differences were observed regarding PVDs, suggesting overlapping functional difficulties across different causes of monocular or reduced vision (unpublished data). These findings support the notion that binocular vision loss disrupts cortical visual input, altering the development of visual pathways and contributing to PVDs beyond VA deficits^13^. Kelly and co-workers reported that loss of binocularity in patients with RB affected the development of visual pathways necessary for face recognition^38^. Depth perception was the most frequently reported difficulty, consistent with the monocular status. As previously discussed, this likely represents a central challenge in daily activities, including judging distances, pouring liquids, or catching objects. Although monocular vision was hypothesized as a cause, excluding individuals with PVD limited to depth perception still revealed a significant difference compared to healthy controls. Similar, three participants reported problems solely with simultaneous perception (difficulty identifying objects within visually complex or cluttered scenes) and excluding them still yielded significant differences compared with healthy controls, underscoring its clinical relevance even among individuals without binocularity. Simultaneous perception might appear less intuitive in individuals with monocular vision, it refers to the ability to process and integrate multiple visual stimuli at once, rather than to binocular fusion. This function can be affected independently of stereopsis and therefore remains a relevant domain of perceptual visual dysfunction. Impairments in movement perception may be particularly relevant for bilateral RB survivors, as retinal damage can interfere with detecting approaching objects or engaging in activities such as ball games. Interestingly, none of our bilateral participants reported difficulties in this domain, suggesting that compensatory strategies may mitigate some of these challenges. Orientation and recognition difficulties were reported by only one participant, which limits generalization, but these problems could have important implications for daily navigation and independence.

PVDs among these patients likely arise from an interplay between disrupted visual pathway maturation, reduced VA, monocular vision, comorbidities, and potential effects of received chemotherapy treatment. In our study, older individuals reported more PVDs which may reflect the balance between increasing social and environmental demands that accentuate visual difficulties, and compensatory strategies developed over time, that can mask or mitigate them. Similarly, a longitudinal study in individuals with the neurodevelopmental disorder FASD indicate that PVDs can persist from childhood into adulthood, with manifestations shifting across ages^37^. The precise mechanisms remain unclear but given the complexity and heterogeneity of visual problems in individuals with RB, in-depth interviews appear particularly valuable for identify the specific vision-related challenges these patients face. Collectively, these findings underscore the need for a comprehensive clinical approach to visual assessment that extend beyond standard acuity measures. Given the high prevalence of PVDs observed in our cohort, a comprehensive clinical evaluation for CVI, including neuropsychiatric assessment in collaboration with an ophthalmologist or orthoptist, would be valuable for understanding functional vision challenges in this population. Such an approach could more accurately capture the complex functional visual difficulties experienced by survivors and guide individualized strategies for care, rehabilitation, and support.

### Strengths and limitations

A strength of the study was the continuity of care provided by a single anaplastologist, which ensured consistent management and follow-up of ocular prostheses. This likely reduced variability in factors such as comfort, cosmetic appearance, and prosthesis function, factors that could otherwise confound quality of life outcomes. However, this study has several limitations. First, the small sample size, while reflective of the rarity of the condition, may have limited the statistical power to detect significant differences and reduced the generalizability of the findings. We acknowledge that the wide age range of participants introduces heterogeneity in visual experience and functional outcomes. Although age-appropriate assessments were applied, developmental and life-stage differences may still have influenced some results. In addition, the small number of age- and sex-matched controls for PVD comparisons may introduce selection bias. While age- and sex-matching was prioritized to enable meaningful comparisons with the RB cohort, these findings should be interpreted cautiously. Additionally, two participants with severe comorbidities reported both PVDs and low HR-QoL, which likely reflect the impact of their underlying medical conditions and may have influenced the overall findings. The inclusion of three patients with bilateral disease may also have affected HR-QoL and PVD outcomes but also contributed to a broader representation within the cohort. Furthermore, not all questionnaires were completed independently under clinician supervision, raising the possibility that responses, from younger participants, were shaped by joint discussions with parents. Children with reduced BCVA in our study consistently scored lower across multiple PedsQL domains, highlighting the relevance of visual function to everyday functioning. The inclusion of a vision-specific QoL instrument could have allowed for a more nuanced understanding of the impact of visual deficits. Future studies should consider combining both general and vision-specific QoL instruments to more comprehensively assess the multidimensional impact of visual impairment among RB survivors. Despite its limitations, this study offers novel insights and is, to our knowledge, the first to examine how children and young adults with RB, treated with ocular protheses, interpret and understand their visual processing by exploring PVDs and their relationship with visual function and HR-OoL.

## Conclusions

Our findings suggest that enucleated RB survivors generally report HR-QoL comparable to that of healthy individuals. However, having subnormal BCVA negatively affected all HR-QoL domains, particularly those related to school functioning. Although PVDs were not directly linked to reduced HR-QoL, their significantly higher prevalence among survivors, regardless of BCVA, underscores the need to assess functional vision beyond VA alone to ensure appropriate support. While the small sample size and cross-sectional design warrant cautious interpretation, the findings highlight the importance of integrated, multidisciplinary care. Such care should include both general and vision-specific QoL assessments, regular ophthalmologic follow-up with attention to PVDs, and individualized educational support. This approach may promote improved long-term well-being and functional outcomes for RB survivors, better equipping them to manage the challenges of daily life and supporting the development of patient-centred survivorship care guidelines.

To further explore the lived experiences underlying these findings, we intend to conduct in-depth qualitative interviews with RB survivors and their parents as a next step. This complementary approach may yield a more nuanced understanding of the functional and psychosocial challenges they face, ultimately informing the development of more personalized and effective strategies for long-term survivorship care within paediatric oncology.

## Supplementary Information

Below is the link to the electronic supplementary material.

Supplementary Material 1

## Data Availability

Due to ethical reasons, data are available from the corresponding author upon reasonable request.

## Abbreviations

A/M: Anophthalmia/microphthalmia
BCVA: Best-corrected visual acuity
CVI: Cerebral visual impairment
FASD: Fetal Alcohol Spectrum Disorder
HR-QoL: Health-related quality of life
LE: Left eye
LogMAR: Logarithm of the minimum angle of resolution
PedsQL: The Paediatric Quality of Life Inventory
PVD: Perceptual visual dysfunction
QoL: Quality of life
RB: Retinoblastoma
RE: Right eye
SD: Standard deviation
VA: Visual acuity
VI: Visually impaired
