# Supplementary material for: Health-related quality of life and visual function in retinoblastoma survivors with ocular prostheses: a cross-sectional study
Source: Sci Rep. 2026 May 14;16:15174. doi: 10.1038/s41598-026-52270-8 (PMC13176331; doi:10.1038/s41598-026-52270-8)
Supplement: Supplementary file 1 — Supplementary Material 1 [file 41598_2026_52270_MOESM1_ESM.docx]

**Questionnaire Regarding Perceptual Visual Dysfunctions (PVDs)**

**Name:** ......................................................
**Personal ID number:** ......................................................
**Date:** ......................................................

Does the above person have difficulties with:

1. Recognizing people? ................................................................................................................
   .....................................................................................................................................................
2. Recognizing people in photographs? .......................................................................................
   .....................................................................................................................................................
3. Recognizing objects? .................................................................................................................
   .....................................................................................................................................................
4. Naming colors? ..........................................................................................................................
   .....................................................................................................................................................
5. Finding their way home? ...........................................................................................................
   .....................................................................................................................................................
6. Finding their way at preschool/school/work? .........................................................................
   .....................................................................................................................................................
7. Orienting themselves in new environments? ..........................................................................
   .....................................................................................................................................................
8. Judging distances and differences in height? ........................................................................
   .....................................................................................................................................................
9. Seeing moving objects? .............................................................................................................
   .....................................................................................................................................................
10. Seeing objects while they themselves are moving quickly? ...............................................
    .....................................................................................................................................................
11. Finding objects on a patterned carpet? ................................................................................
    .....................................................................................................................................................
12. Finding objects in complex pictures? ...................................................................................
    .....................................................................................................................................................
